# Color of Pan Trap Influences Sampling of Bees in Livestock Pasture Ecosystem

**DOI:** 10.3390/biology10050445

**Published:** 2021-05-19

**Authors:** Roshani S. Acharya, Timothy Leslie, Emily Fitting, Joan Burke, Kelly Loftin, Neelendra K. Joshi

**Affiliations:** 1 Department of Entomology and Plant Pathology, 217 Plant Sciences Bldg., University of Arkansas, Fayetteville, AR 72701, USA; rsharmaa@uark.edu (R.S.A.); emily.fitting@maine.edu (E.F.); kloftin@uada.edu (K.L.); 2 Department of Biology, Long Island University, 1 University Plaza, Brooklyn, NY 11201, USA; Timothy.Leslie@liu.edu; 3 USDA-Agricultural Research Service, Booneville, AR 72927, USA; joan.burke@usda.gov; 4 Current address: School of Marine Sciences, University of Maine, Orono, ME 04469, USA

**Keywords:** pollinators, pan traps, pasture ecosystem, bees, bee vision, sampling method

## Abstract

**Simple Summary:**

Pollination is important for fertilization, setting fruits, seed development and the continuation of the life cycle of plants that eventually provide food for humans, livestock and wildlife. Agronomic practices, use of pesticides, lack of diverse flowering plant species, introduction of invasive plants, loss of habitat, climate change and disease have all led to the decline of important pollinator species. Decline of insect pollinators has increased the importance of accurately monitoring pollinator diversity and abundance over time. Sampling techniques using different color traps are used to sample bees and other insects, but their utility and effectiveness in different ecosystems still need to be determined. In this study, we examined four different colors of pan traps (blue, green, yellow, and purple) for their utility in sampling bees in a livestock pasture ecosystem consisting of native forage species. We analyzed the relative abundance, richness, similarity, and community assemblage patterns associated with aforementioned colors. We found that the blue color traps were the most attractive to bees and were effective for sampling bees in a livestock pasture ecosystem. Purple color traps were the second most effective, followed by yellow and green color traps.

**Abstract:**

The decline in insect pollinators has increased the importance of accurately monitoring pollinator diversity and abundance over time. Sampling techniques include the use of passive insect traps such as pan traps, yet there is still discussion over their utility and effectiveness in different ecosystems. The objective was to examine four different colors of pan traps (blue, green, yellow, and purple) for their utility in sampling bees in native forages rotationally grazed by sheep and to compare the relative abundance, richness, similarity, and community assemblage patterns among the four trap colors. Most bees were from the Halictidae family (89%). The most abundant species were *Lasioglossum imitatum* (42.2%), *Augochlorella aurata* (8.3%), *L. subviridatum* (6.8), *Agapostemon texanus* (6.4), and *L. birkmani* (4.1%). Blue color traps exhibited the highest rates of bee capture and species accumulation. Purple and yellow colored traps were moderately effective in capturing bees, while the green color pan traps were least effective. Similarly, observed and extrapolated species richness was highest in blue trap, followed by purple, yellow, and green. Notably, the blue trap captured the highest number of unique species, followed by purple, yellow and green traps. Considering the total number of insects collected (including bees and other insects), yellow and green traps captured a significantly higher number of insects than other colored traps. The light reflectance from blue, purple, green and yellow pan traps had peaks at ~450, 400, 550, and 600 nm, respectively. Since different insects respond to different light intensities, wavelengths, and reflectivity, these results could be used to guide future trapping protocols targeting certain insect groups in livestock pasture and similar ecosystems.

## 1. Introduction

Bees are the most important pollinators for fruits, vegetables, nuts, forages and many other economically important crops as well as wild flowering plants. However, in recent years, intensification of agriculture has led to a decrease in foraging resources of bees and their potential nesting sites [1]. Such intensification has negatively impacted the environment and subsequently decreased bee populations [2]. For instance, declines in bumblebees (*Bombus* spp., Hymenoptera: Apidae) have been linked to agricultural intensification [3]. Recent declines in pollinators have raised concerns not only for their conservation, but also for the potential of pollination deficits in various ecosystems [4]. 

Estimates of pollinator abundance and diversity in a specific region depend on the frequency of monitoring and sampling procedures. Different types of sampling methods, including pan traps, may be used for sampling bees. Pan traps are an effective and commonly used technique for trapping insect pollinators, especially bees [5]. Additionally, pan traps can potentially allow researchers to monitor pollinator population across space and time as use of the traps does not negatively impact bee populations when sampling occurs every other week [6].

Pan traps, also known as water traps, are commonly used for sampling agricultural insect pests [7] as well as other arthropods (such as parasitoid wasps) in different ecosystems. Pan traps are passive traps that are cheaper and more effective in capturing a large number of Hymenopterans than other sampling methods such as malaise trap [8]. Pan traps do not suffer from observer bias, which may occur when using active net sampling [5]. Although pan traps may not always provide an accurate representation of the bee fauna in a particular area [9], they still remain one of most common methods for sampling bees as they are a cost-effective and efficient way to sample from a large area in a short period of time [7]. Pan traps are effective in all geographical locations, agricultural lands, and semi-natural habitats, even when few flowers are available [10].

The intensity of the light reflected differed by color of traps and thus affects the number of bees and other pollinator species that are attracted toward the traps [11,12]. When sampling, considering the color vision of hymenopteran insects, such as bumblebees and honeybees, it is important because it impacts their attraction toward different colored traps [13]. Bees are able to identify dissimilar colors and discriminate color textures (fine or course), although such ability may vary by species. In the case of honeybees, visual angle depends on stimulated photoreceptors for coding the color information [13]. Color discrimination senses in bumblebees are more poorly developed than in honeybees; however, bumblebees observe stimuli with smaller visual angle in comparison to honeybees [14]. Color intensity and chromaticity of illumination provide contextual cues that direct bees toward the source [15]. Bees also use their olfactory signals if the source is 30 cm away and visual cues when they are nearer to flowers [16]. 

Measurements of abundance, species richness and diversity of insect pollinators collected by pan trapping may be influenced by the color of the pan traps being used and the type of ecosystem in which the sampling occurred. For example, the high reflective index of white or yellow pan traps attracted the greatest richness of anthophilic insects in the lowlands of the Cape Floristic Region in South Africa [11]. Between yellow and white colored pan traps, yellow traps were found to be more efficient in attracting a higher number and diversity of bees in open fields, riverside habitats, forests and roadside verges in Australia [17]. Overall pollinator abundance and diversity was also higher in yellow traps compared to white and blue traps in the Yellow River region of China [18]. Yellow traps have also been shown to be more effective at attracting both wasps [19,20] and hoverflies [21] than other trap colors. Conversely, blue pan traps have been reported to be highly effective at trapping bees in a variety of ecosystems, including fruit orchards [12], savannas [20], and forested ecosystems [8]. Whether pan traps are fluorescent or non-fluorescent may also affect insect capture rates [22]. In addition to diversity measures, the sex ratios of bees in samples collected in pan traps may vary based on the color of pan trap, and differences in color preferences between males and females can vary among species [7,23].

Abundance, diversity, and sex of bees and other pollinators could vary with color and light reflectance of the colored pan trap because the color vision of hymenopterans, such as bumblebees and honeybees, impacts their attraction to the source. Thus far, the majority of studies in this field have used blue, yellow and white-colored pan traps. However, other colored traps within the visible light spectrum for bees, such as green and purple, may also attract bees, and such visual attractancy of several other colors to bees is yet to be documented. Therefore, the main goal of this study was to investigate whether differently colored pan traps—including commonly used blue and yellow traps, and less commonly used green and purple traps—impact measurements of wild bee abundance and diversity, as well as bee assemblage patterns, in a livestock pasture ecosystem. The findings of this study would be useful in selecting the most appropriate type of colors for pan trapping bees in pastures, which are different than other agro-ecosystems in many ways. 

## 2. Materials and Methods

### 2.1. Site Description

This study was conducted during July and August of the 2018 field season at the research farm of the USDA-ARS Dale Bumpers Small Farms Research Center in Booneville, Arkansas (35.09° N, 93.95° W). The soil of the site is characterized as Leadvale silt loam (fine-silty, siliceous, semiactive, thermic Typic Fragiudults) (https://websoilsurvey.sc.egov.usda.gov, accessed on 12 May 2021). Average temperatures at the research station during July and August of 2018 were 27.0 and 25.2 °C. The site received 96.9 mm and 151 mm rainfall during July and August, respectively.

### 2.2. Study Site: History and Preparation

Pasture management prior to the study included herbicide treatment: Roundup^®^ (41% glyphosate; Monsanto, St. Louis, MO, USA; 0.764 L/ha) in June, July, September, October of 2016 and January of 2017, and Outrider (75% Sulfosulfuron; Monsanto, St. Louis, MO, USA; 0.016 L/ha) in September 2016 using a Continental Belton cluster nozzle sprayer (Continental Belton McAlester, SR: A44117, Oklahoma City, OK, USA). The site was burned in September 2016 and prepared with a tiller (Maschio Gaspardo North America Inc., SC 300, DeWitt, IA, USA) and rolled using a 12′ Big Guy Roller (Grahl Manufacturing, Republic, MO, USA) in October 2016. Mixtures of Tallgrass Inexpensive Seed Mix (TGI; Prairie Moon Nursery, Winona, MN, USA; 5.4 kg/acre), Buck’s Hangout (BH; Hamilton Native Outpost, Elk Creek, MO, USA; 5.89 kg/acre) and Tallgrass Exposed Clay Subsoil Mix (TGE; Prairie Moon Nursery; 10.88 kg/acre) were then planted in February 2018. Plant species composition in these seed mixes at the time of sowing are available on their respective supplier websites.

Plots were grazed by sheep in late June of 2018 to provide forage to the animals, reduce weeds (mixed non-native grass species) that were palatable to sheep, and maintain native grasses in vegetative state. By early July, there were estimated to be at least three native flowering species per plot, even after being grazed by sheep. The most common flowering species visually documented during the sampling period were *Verbena hastata*, *Pycnanthemum verticillatum*, *Echinacea pallida*, *Coreopsis* spp., *Monarda fistulosa*, *Aster novae-angliae*, *Verbena* spp., *Cichorium intybus*, and *Daucus carota.* The adjacent landscape of the study site was mainly comprised of pastureland as well as some natural areas with unmanaged habitats and woods.

### 2.3. Pan Traps and Sampling

**Figure 1:**
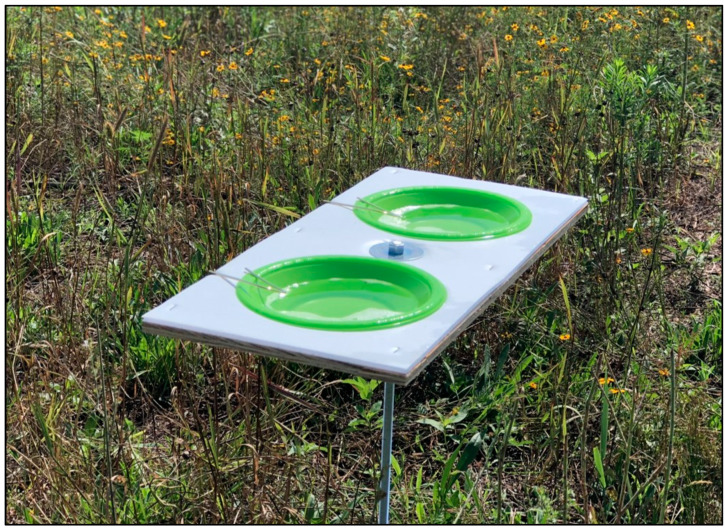
A colored pan trap platform used in the study. Picture by N. Joshi.

Four transects (~100 m long, one per plot) of four elevated pan trap platforms were established at the study site comprised of four plots (0.4 ha each). Each individual platform contained two pan traps of the same color. Within each transect, four trap colors (yellow, green, blue, and purple) were deployed, and the order of the platforms was randomly assigned. Traps consisted of 354.88 mL fluorescent colored plastic bowls with UV reflectance, in which blue (color: bright royal blue 105; Festive Occasion, East Providence, RI, USA), yellow (color: school bus yellow; Touch of Color, Creative Converting, Clintonville, WI, USA), green (color: fresh lime; Touch of Color, Creative Converting, Clintonville, WI, USA), and purple (color: purple; Touch of Color, Creative Converting, Clintonville, WI, USA) bowls were used. Sampling began in early July and samples were collected four times per week until mid-August. Trap platforms were placed 25 m apart in transects, and the distance between transects was 15 m. The nearest trap to the fences that separated study plots was 8 m from the boundary. Pan trap platforms were placed ~1.25 m above the ground to match the height of the canopy of flowering plants in the pasture. For each sampling event, two-thirds of each bowl were filled with soapy water (Figure 1). In order to prepare soapy water, a few drops of unscented liquid dishwashing detergent were mixed with 3.785 L of tap water. Insect samples were collected from the traps after approximately a 24 h period. Traps were set up in the morning at 7:00 a.m. to be collected on the next day around the same time.

Samples collected from each platform (two pan traps) were placed in plastic vials containing 70% ethyl alcohol before they were transported to the laboratory. In the laboratory, insects were air dried to remove ethanol, sorted, pinned, boxed and shipped for identification. Samples were identified to the species level by Drs. D. Biddinger (Department of Entomology, Penn State Fruit Research and Extension Center, Biglerville, PA, USA) and R. Jean (Senior Entomologist, Environmental Solution & Innovations, Inc., Indianapolis, IN, USA). Due to taxonomic difficulties in identifying all insects to species level, only bees were considered for analysis at species level.

### 2.4. Light Reflectance Analysis of Pan Traps

All colored pan traps were analyzed for their light reflectance characteristics at the Department of Chemistry and Biochemistry, University of Arkansas, Fayetteville, USA. For this purpose, a small, square-shaped piece (2 cm^2^) of each colored bowl or trap was kept inside the incident light window of spectrophotometer where light was reflected from the sample on the detector for 200 sec. The detector was attached to a barium sulfate coated sphere with a 60 mm integrating sphere. Total reflectance of each color of pan trap was recorded within 190–1600 nm range of spectrum using a JASCO V-780 spectrometer (JASCO Corporation, Easton, MD, USA). Intensity of light passing through the sample was recorded by using Spectra Manager^TM^ suite, spectroscopy software connected with Windows 7 pro (64-bit) operating system. The light reflectance and wavelength of each sample were recorded and analyzed. The baseline measurement was taken at the beginning.

### 2.5. Data Analyses

Measures of bee abundance, richness, similarity, and community assemblage patterns were compared among the four trap colors. The overall effect of trap color on bee abundance and total insect abundance was tested using analysis of variance (ANOVA) in JMP. A post hoc Tukey test was then conducted to identify significant differences among trap colors. For each trap color, data were summed within each of the 18 sampling dates. These abundance values were square-root transformed prior to analysis to addressing the right-skewness (i.e., preponderance of low values) in the original dataset.

The species richness of bees was compared among trap colors by developing sample-based and individual-based rarefaction curves in EstimateS v7.5 [24]. Rarefaction curves depict interpolated species accumulation through iterative resampling from the species-by-sample abundance data matrix. Rarefaction curves allow for direct comparison of expected species richness among treatments at a standardized number of samples or individuals collected. Differences in species accumulation rates were determined based on non-overlapping 95% confidence intervals. A Chao1-richness estimator was also used to plot extrapolated species accumulation curves based on the number of rare species in the samples. In addition, incidence-based (Sorensen Classic) and abundance-based (Chao-Sorensen raw abundance-based) similarity measures were calculated in EstimateS v7.5 [24] for each pairwise combination of trap colors in order to characterize the extent of species similarity among traps colors. The number of unique species found in each trap color was also reported. 

To examine the community assemblage patterns associated with trap color, a constrained ordination was conducted using Canoco v4.5 [25]. Trap colors, coded as dummy variables, were used as environmental predictor variables. Since a large number of bee species were found to be singletons or doubletons, bee data were aggregated at genus level. For each combination of trap color and sampling date, counts of bees within each bee genera were used as response variables. Following a detrended correspondence analysis (DCA) to evaluate data structure, a canonical correspondence analysis (CCA) was used to generate orthogonal axes representing models explaining the greatest amount of variance in the species data. Species data were squared root transformed and centered and standardized for analysis. The significance of trap color was assessed through Monte Carlo permutations (*n* = 999) and stepwise forward selection [26]. Biplots were developed in CanoDraw [26] to visualize associations between bee genera and trap colors.

## 3. Results

### 3.1. Abundance and Diversity 

**Figure 2:**
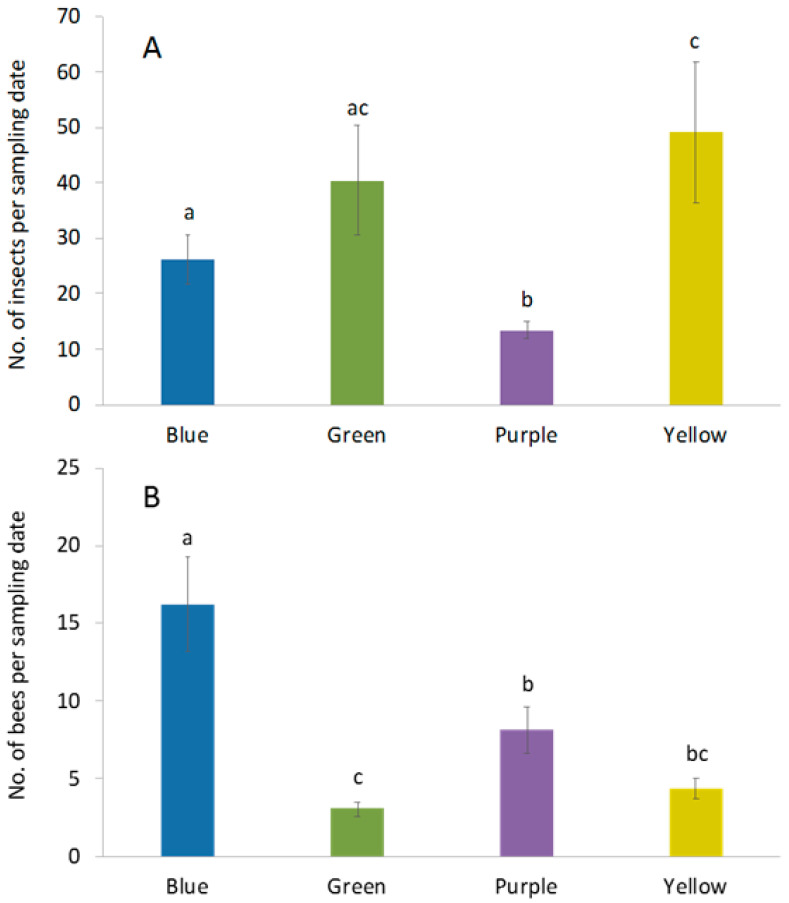
Differences in capture rate (±SE) of (**A**) total insects and (**B**) bees among four pan trap colors in livestock pastures. There were four trap color in each of the 4 plots. Samples were taken 4 times each week for 8 wk. Different lowercase letters indicate significant differences among trap colors (*p* < 0.05).

**Figure 3:**
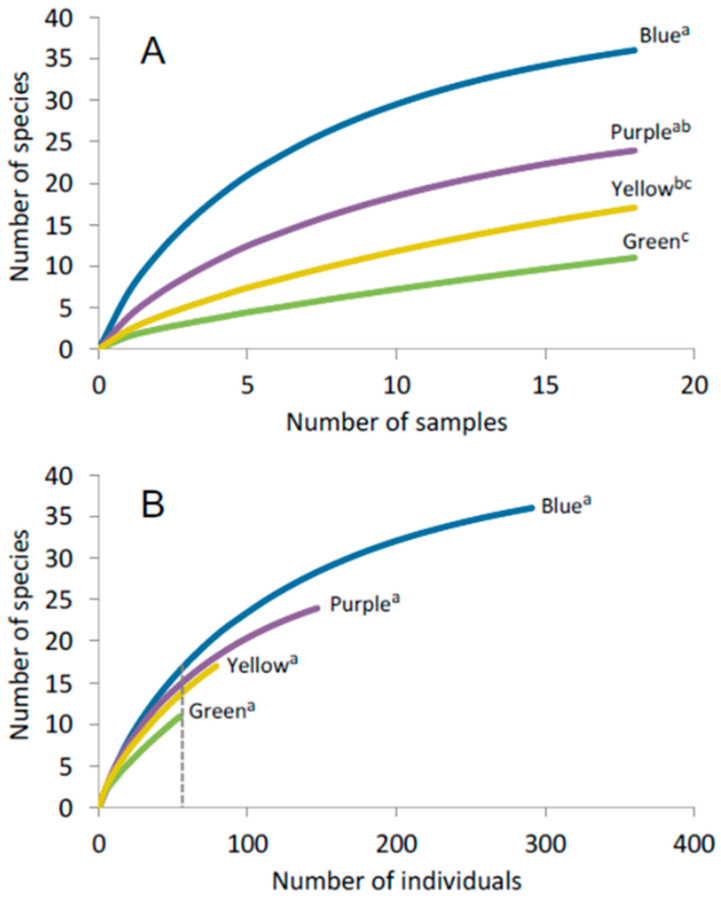
Rarefaction curve showing accumulation of the number of species in relation of the number of samples (**A**) and number of individuals (**B**). Dotted line in (**B**) indicates at what abundance value interpolated richness values were compared. Different superscript letters indicate significant differences among trap colors.

Over the course of the study, 2327 insects were captured in pan traps. Insect capture rates differed among trap colors (F_3,68_ = 4.24; *p* = 0.008). Yellow traps captured the greatest number of insects, followed by green, blue and purple traps, respectively (Figure 2A). Among these insects, a total of 573 bees comprising 44 species from four families were collected (Table 1). Bee abundance (Table 2; Figure 2B) also differed among trap colors (F_3,68_ =12.5; *p* < 0.0001) and was higher in blue pan traps compared to all trap colors (Figure 2B). Purple pan traps had the second highest capture rate followed by yellow and green pan traps (Figure 2B). Similarly, observed and extrapolated (Chao1) species richness was highest in blue traps, followed by purple, yellow and green traps, respectively (Table 2). Sample-based rarefaction curves revealed that species accumulation was significantly higher in blue traps relative to yellow and green traps (Figure 3A). In addition, sample-based species accumulation in purple traps was higher than in green traps (Figure 3A). Conversely, no significant difference in species accumulation could be detected among trap colors using individual-based rarefaction (Figure 3B).

**Figure 4:**
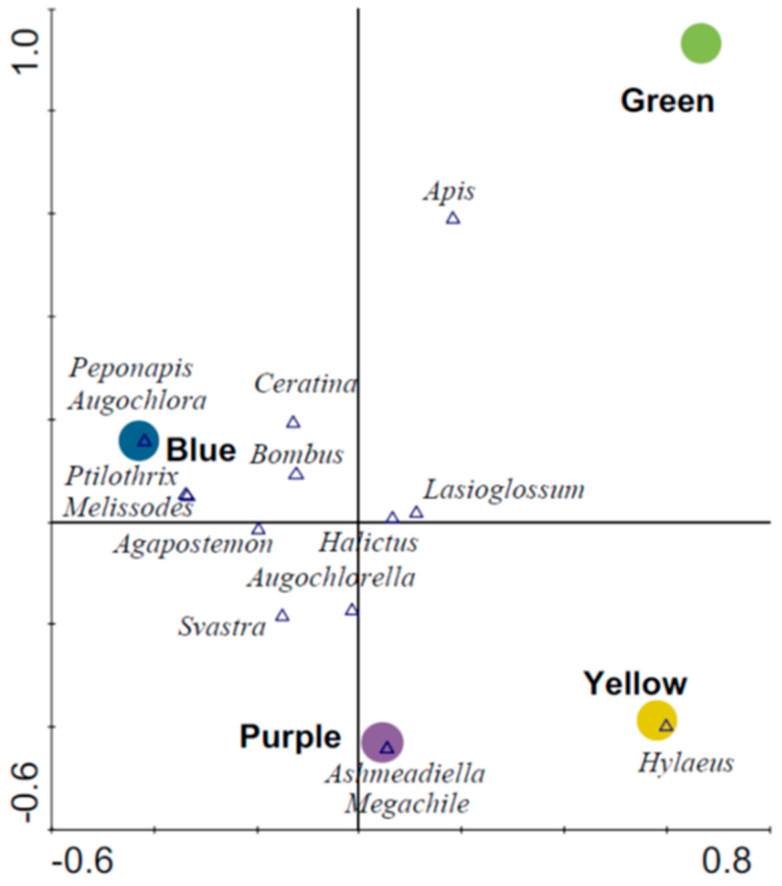
Ordination (CCA) biplot showing associations among wild bee species and different colored pan traps.

Blue traps had the highest number of unique species followed by purple, green and yellow traps, respectively (Table 2). Similarity in species composition based on pairwise comparisons was greatest among blue, purple and yellow traps, whereas green traps had the most dissimilar species composition when compared to other trap colors (Table 2). Ordination revealed distinct bee assemblages associated with blue traps (F = 2.06, *p* = 0.023) relative to other trap colors (Figure 4). This first axis on the biplot explained 3.5% of the species data and 52.3% of the species–environment relation. The secondary (vertical) axis explained an additional 2.1% of species data and 31.0% of the species–environment relation and depicts associations between green traps (F = 1.49; *p* = 0.17) and yellow and purple traps (Figure 4).

Most of the bees recorded in this study were from the Halictidae family (89%). Of the total bees, 50.9% were collected from blue traps, 26.6% from purple traps, 13.8% from yellow traps, and 9.6% from green traps. The most abundant species was *Lasioglossum imitatum* (Smith) (42.2% of total), followed by *Augochlorella aurata* (Smith) (8.3%), *L. subviridatum* (6.8% of total), *Agapostemon texanus* (Cresson) (6.4% of total) and *L. birkmani* (4.1% of total, Table 1).

### 3.2. Light Reflectance

**Figure 5:**
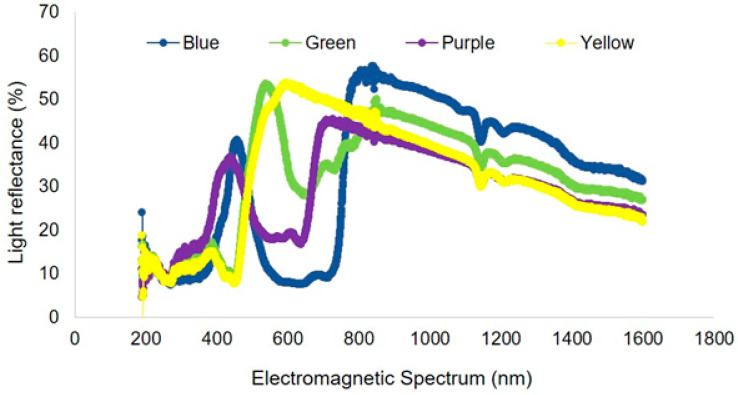
Light reflectance spectrum for four different colors of pan traps used for sampling pollinators and other insect communities.

**Figure 6:**
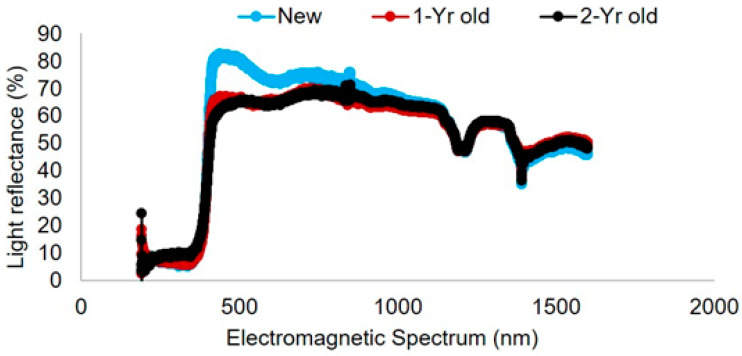
Light reflectance spectrum of the white color base of pan trap platform used in this study. Light reflectance spectrum of new (New), one-year-old (1-Yr old), and two-year-old (2-Yr old) platforms is presented.

Light reflectance curves varied among trap colors (Figure 5). Within the visual spectrum of bees, the blue pan trap had a peak at 450 nm with wavelength ranging from 300 to 500 nm. The frequency of wavelength of purple traps ranged from 200 to 450 nm (with a peak of 400 nm). Green traps had a peak at 550 nm with wavelength ranging from 200 to 600 nm, while yellow traps had a peak around 600 nm with a frequency range from 200 to 600 nm. Traps with higher light reflectance in the 300–500 nm range attracted the most species of bees in this study. White platforms where traps were kept (as shown in Figure 1) had a light reflectance peak at 500 nm with a range of 200–600 nm wavelength (Figure 6).

## 4. Discussion

For the conservation of pollinators, it is necessary to monitor their abundance and diversity in different habitats, ideally in relation to reliable baseline records. For this, effective techniques are needed to trap them across space and time that do not negatively impact their persistence [6]. Although different trapping techniques will undoubtedly contain biases [9], pan traps remain a simple and effective pollinator sampling technique as compared with other methods such as net sampling or malaise traps [5,8,12]. Yet, there remains a need to better understand the effect of trap color and reflectance on measures of abundance and diversity of pollinators collected in different ecosystems.

Among the four different pan trap colors (blue, yellow, green and purple) that were tested in this study, bee capture rates and species accumulation rates were highest in blue pan traps compared to other colors of pan traps in livestock pasture plots. This finding agrees with other studies that have shown blue traps to be particularly attractive to bees [12]. Purple traps were also highly effective. Similarity in community composition between blue and purple traps suggest reduced complementarity; however, the fact that blue and purple traps had ten and four unique species, respectively—and distinct genera groupings revealed by ordination—suggests that including purple traps in field sampling should be considered. Overall, yellow pan traps were most effective to attract the greatest number of insects (of all groups). This difference highlights the importance of trap color selection when monitoring targeted insect groups. Green traps were the least effective of the four trap colors, which is likely due to the predominant green pasture background, resulting in reduced color contrast. However, there could also be some impact of white colored platforms (used to hold pan traps) on overall color contrast and could have influenced attractiveness of pan traps to different species of bees. 

Flower color is a visual clue for detection, recognition, and memorization of food resources for bees, and other anthophilic insects [27]. The intensity of light reflected from traps was dependent on color which affects the number of pollinators, especially bees attracted toward the traps [11]. The visual spectrum of most insects usually includes UV light [27]. In most cases, the visual spectrum of bees has been reported to be in the range of 300 to 630 nm [28]. Specifically, insects in the order Hymenoptera, including bumbles bees and honeybees, are impacted by color vision [13]. In the case of honeybees (*Apis mellifera*), color vision is trichromatic having ultraviolet, blue and green photoreceptors with maximum sensitivity at 350, 440 and 540 nm [29]. 

The differences in capture rates among trap colors in the current experiment can be explained by the visual spectrum of bees and the measured light reflectance. Blue traps had highest light reflectance in the 300–500 nm range, which likely played an important role in attracting different bee species. Likewise, purple traps, which had the second highest rate of bee capture and species, also had the second highest level of light reflectance in the 300–500 nm range. In general, bees discriminate blue, blue-green, violet, and yellow in the spectrum [30]. Although bees have a color spectrum from UV to orange, they may also utilize color contrast to find target objects [31]. For example, white flowers lack color contrast (as perceived by bees) against vegetation and other backgrounds resulting in a neutral color. Bees may thus ignore such flowers or rely on other visual cues to detect them [28]. 

A total of 44 species from 15 genera and four families were collected during July and August in 2018. The number of species documented in the current study is half than that reported by [32] in managed emergent wetlands within the lower Mississippi Alluvial Valley of Arkansas, USA, and one quarter of number of species reported by [33] in the Arkansas River Valley. Most of the bees collected from the current study belong to Halictidae family (89%) which is similar to other findings in which pan traps were used for collecting pollinators in earlier studies [34] in Oregon and [35] in Texas. The most abundant species in the current study consisted of *L. imitatum* (42.2%), followed by *Augochlorella aurata* (8.3%), *L. subviridatum* (6.8%), *Agapostemon texanus* (6.4%) and *L. birkmani* (4.1%). Surprisingly, in the study by [32], there was no report of *L. imitatum*, *L. subviridatum* and *L. birkmani* though both of the studies were conducted in the same state in Arkansas in the same season (during months of July through August), but in a different landscape and ecosystem. In another study conducted in a nearby location, *Lasioglossum* spp. (Hymenoptera: Halictidae) was the most prevalent genus [33]. The most dominant genera reported were *Lasioglossum* spp., followed by *Megachile* spp., *Augochlorella* spp, *Bombus* spp., and *Melissodes* spp. 

Pollinator species richness and functional diversity, as well as species-wise distribution, in livestock pastures vary during the season [36]. The current study was performed during the latter half of the season (mid-July to mid-August) whereas other studies were performed throughout the entire season [35,36], and such differences in the timing and duration of sampling could be the reason we documented fewer bee species in comparison with previous studies. In addition, the collection method in the current study (pan trap) was different than that used in previous studies (deep bowl trap that could hold more pollinators [35]; vane trap [36]) and thus could have missed capturing diverse species. However, the major dominating bees (*Bombus*, *Halictus*, *Lasioglossum*, and *Melissodes* spp.) during the July–August period in the current study largely overlapped with previous studies [35,36]. Consistent with the current study, [35] reported 76% of Hymenoptera followed by lesser percentage of Diptera and Lepidoptera during the summertime in pastures of southern US.

Abundance of bees in livestock pasture depends on numerous factors such as plant species composition and architecture, soil and microclimatic characteristics, and grazing intensity [34]. Use of herbicides could decrease species richness by 71% [37]. In the current study, there was no application of herbicides for up to 3 years before the sampling, and it is not likely that herbicide residues could have impacted species diversity in the current study. Trap height relative to height of canopy could impact survey of pollinators. Little has been published on pan trap height in livestock or native pastures. A study in high bush blueberries showed that traps mounted at one third the height of the canopy captured significantly higher numbers of bees than traps at higher or lower heights [38]. Traps used in the current study were relatively higher than the forage height, thus may not have been optimal for bee capture, and further research in this regard is warranted.

## 5. Conclusions

This study revealed that blue pan traps were the most effective pan trap color for sampling bees in a livestock pasture ecosystem. Purple traps were the second most effective, followed by yellow and green traps. These findings are supported by the reflectance value of the color trap (with or without soapy water) and the known visible light spectrum of bees. Notably, yellow and green traps were the most effective traps for sampling insect communities in general but not for sampling wild bees. In addition, the results show that livestock pasture ecosystems that include native forages can support a wide variety of bees, which—in combination with grazing and management intensity—should be considered in pollination conservation schemes in agricultural landscapes.

## Figures and Tables

**Table 1:** Bee species diversity (family, genus, and species) collected using different color pan traps (blue, green, purple, and yellow) in Arkansas livestock pasture ecosystem in 2018.

Diversity of Bees			Pan Trap Color			
Family	Genus	Species	Blue	Green	Purple	Yellow
Apidae	*Apis*	*mellifera*	x	x		
	*Bombus*	*griseocollis*	x		x	x
	*Bombus*	*pensylvanicus*	x	x		
	*Ceratina*	*strenua*	x	x	x	
	*Ceratina*	*calcarata*	x	x		
	*Melissodes*	*niveus*	x		x	
	*Melissodes*	*veroninae*	x			
	*Melissodes*	*bimaculata*	x			
	*Melissodes*	*communis*	x		x	
	*Melissodes*	*comptoides*	x			
	*Peponapis*	*timberlakei*	x			x
	*Ptilothrix*	*bombiformis*	x		x	x
	*Svastra*	*atripes*			x	
	*Svastra*	*obliqua*	x		x	
Colletidae	*Hylaeus*	*rudbeckiae*				x
Megachilidae	*Megachile*	*brevis*			x	
	*Ashmeadiella*	*floridana*			x	
Halictidae	*Agapostemon*	*texanus*	x	x	x	x
	*Agapostemon*	*splendens*	x		x	
	*Agapostemon*	*sericeus*	x		x	
	*Augochlorella*	*aurata*	x		x	x
	*Augochlorella*	*persimilis*	x			x
	*Augochlora*	*pura*	x			
	*Halictus*	*rubicundus*	x		x	x
	*Halictus*	*confusus*	x	x	x	x
	*Halictus*	*ligatus*	x		x	x
	*Halictus*	*parallelus*	x	x		
	*Lasioglossum*	*imitatum*	x	x	x	x
	*Lasioglossum*	*disparile*		x	x	
	*Lasioglossum*	*versatum*	x		x	x
	*Lasioglossum*	*coreopsis*	x			x
	*Lasioglossum*	*nr versans*	x		x	
	*Lasioglossum*	*birkmanni*	x		x	x
	*Lasioglossum*	*subviridatum*	x	x	x	x
	*Lasioglossum*	*foxii*			x	
	*Lasioglossum*	*sopinci*				x
	*Lasioglossum*	*paraforbesii*	x			x
	*Lasioglossum*	*athabascence*	x			
	*Lasioglossum*	*tegulare*	x		x	
	*Lasioglossum*	*pectorale*	x			
	*Lasioglossum*	*trigeminum*	x			
	*Lasioglossum*	*callidum*	x			
	*Lasioglossum*	*zephyrum*		x		
	*Lasioglossum*	*hitchensi*	x			

**Table 2:** A comparison of bee diversity measures among four colors of pan traps deployed in Arkansas livestock pastures.

		Pan Trap Color			
		Blue	Green	Purple	Yellow
**Abundance**		291	55	147	79
**Richness (observed)**		36	11	24	17
**Richness (extrapolated; Chao1) ^1^**		45	22	28	22
**Number of unique species**		10	1	4	2
**Similarity Indices ^2^**	Blue	1			
	Green	0.38 (0.70)	1		
	Purple	0.63 (0.89)	0.34 (0.75)	1	
	Yellow	0.53 (0.87)	0.36 (0.76)	0.59 (0.90)	1

^1^ Rounded to nearest whole number. ^2^ Sorensen classic and Chao–Sorensen raw abundance-based (in parentheses) similarity indices.

## Data Availability

The datasets from this study are available from the corresponding author on reasonable request.
